# A Multifunctional Shape-Adaptive Bilayer Hydrogel for Acute Hemostasis, Wound Repair, and Insect Bite Defense

**DOI:** 10.3390/gels12040347

**Published:** 2026-04-21

**Authors:** Rongyan He, Wenhui Yan, Qiuyu Cao, Chun Zhang, Yuxiu Ye, Yao Chen, Shaoxian Wu, Fei Han, Sulan Luo

**Affiliations:** 1 Guangxi Key Laboratory of Special Biomedicine, School of Medicine, Guangxi University, Nanning 530004, China; herongyan@gxu.edu.cn (R.H.); xzkbk5683@163.com (C.Z.); 18877548173@163.com (Y.Y.); cy38514@163.com (Y.C.); wsx14050@163.com (S.W.); 2 The Key Laboratory of Biomedical Information Engineering of Ministry of Education, School of Life Science and Technology, Xi’an Jiaotong University, Xi’an 710049, China

**Keywords:** wound dressing, insect bite defense, hemostasis, shape-adaptive hydrogel

## Abstract

Fieldwork carries a high risk of irregular, non-compressible traumatic wounds, which often initiate a vicious cycle of “traumatic bleeding-insect bite-secondary infection”. Conventional dressings cannot combine rapid hemostasis with physical protection against venomous insects, creating an urgent demand for multifunctional field trauma dressings. To solve this problem, this study developed a shape-adaptive bilayer hydrogel that concurrently provides rapid hemostasis, promotes wound repair, and acts as a robust physical barrier. The hydrogel adopts a layered design: the bottom layer (PPTY) achieves autogelation within 3 s upon blood contact, while the top armor protective layer (AP) withstands pressures up to 942 kPa. By incorporating chitosan and sodium citrate into the AP precursor solution, the hydrogel achieved in situ formation within 50 s and developed a stable self-renewing armor layer. The tightly bonded bilayer showed complementary functions. In rat models of femoral artery puncture and tail vein bleeding, PPTY-AP hydrogel significantly reduced blood loss and shortened hemostasis time. Moreover, the hydrogel demonstrated excellent tissue adhesion and moisture retention capacity, promoting full-thickness skin wound healing. This multifunctional, rapidly deployable hydrogel presents a promising solution for field trauma management and offers a new design paradigm for advanced wound dressings.

## 1. Introduction

Personnel in field operations, such as remote area exploration, natural disaster rescue, and extreme environment expeditions, face a high risk of traumatic injuries. These wounds are often irregular in shape and difficult to manage with compression hemostasis. Uncontrolled bleeding remains a leading cause of preventable death in trauma, underscoring the critical need for rapid and effective hemostatic strategies [1,2,3,4,5]. Furthermore, open wounds in the wild are prone to contamination and face unique environmental challenges: blood and other body fluids exuding from the wound release volatile signal molecules, which are potent chemical attractants for hematophagous arthropods like mosquitoes and flies [6,7]. These insects inflict secondary tissue damage, cause localized inflammation and pain, and serve as vectors for pathogens responsible for diseases including malaria, dengue, and Zika virus [6]. Furthermore, they can introduce pathogens into the wound, potentially inducing serious secondary infections like cellulitis, lymphangitis, and sepsis [8,9]. Consequently, this vicious cycle of “skin trauma and bleeding—insect bites—secondary infection” prolongs the inflammatory phase, severely impedes the natural healing process, and can even be life-threatening [8]. An ideal field wound dressing must achieve rapid hemostasis, support healing, and provide a durable physical barrier that is, for example, able to defend against insect bites and environmental pathogens.

Existing wound dressings do not adequately meet these combined demands. Traditional materials such as gauze and bandages have limited tissue adhesion and generally require continuous external compression, which restricts their effectiveness in irregular or incompressible wounds [1,2,10,11]. Hydrogels with their inherent biocompatibility, and hydration capacity, are promising candidates for advanced dressings [12,13,14]. The development of shape-adaptive hydrogels has promoted the development of wound management and tissue regeneration. Joshi et al. developed a 4D-printed conductive hydrogel with programmable shape transformation via differential swelling for biomimetic nerve conduits, demonstrating that structural design and stimulus responsiveness endow hydrogels with tissue adaptability and self-folding ability for flexible bioelectronics and regenerative medicine [15]. Furthermore, shape-adaptive hydrogels can fit irregular tissue defects and adapt to tissue deformation, improving their interface interaction with biological tissues and thus boosting hemostasis, repair, regeneration, and other therapeutic effects [16,17]. Military hemostatic agents based on zeolite and chitosan (such as QuikClot^®^, Celox^®^) and shape-adaptive self-gelling hydrogels adapt well to irregular, incompressible wounds, avoid adhesion issues of traditional dressings, and provide a favorable microenvironment for wound repair [1,16]. PPTY self-gelling powder gels within 3 s on blood contact, with strong tissue adhesion and excellent hemostatic and wound-healing effects [16]. However, conventional PPTY hydrogels are mechanically weak and unable to form an effective physical barrier, limiting field applications. Hydrogels with protective physical barrier properties thus remain underdeveloped.

Recent advances in toughening hydrogels offer potential solutions. For instance, Zhang et al. incorporated sodium lignosulfonate into PVA hydrogel, forming silver nanoparticles via microphase separation to achieve a toughness of 50.7 MJ/m^3^ [18]. Zhao et al. developed an armor-protected hydrogel inspired by starfish, where in situ sodium acetate crystallization forms a surface hard layer with a soft interior, offering flexibility, self-repairability and effective physical barrier protection [19]. However, the material lacks inherent hemostatic activity and requires 30 min of UV curing, limiting its field emergency application.

**Figure 1:**
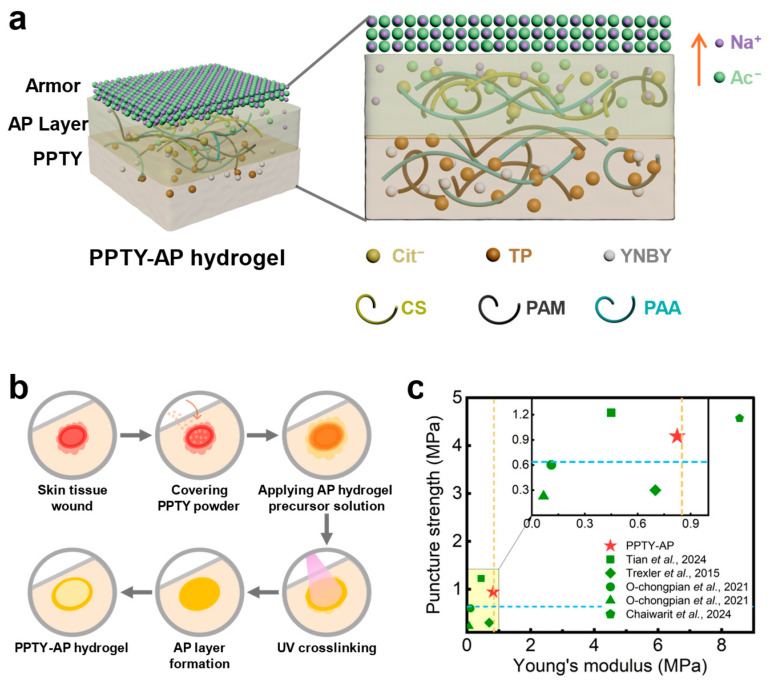
Schematic diagram and mechanical properties of PPTY-AP hydrogel structure. (**a**) Schematic diagram of PPTY-AP hydrogel; (**b**) The application of PPTY-AP hydrogel. (**c**) Comparison of Young’s modulus and puncture resistance between PPTY-AP hydrogel and reported wound dressings. The blue dashed line indicates the bite force of venomous insects, while the yellow dashed line represents the Young’s modulus of adult human skin [17,24,25,26].

To address the limitations of traditional single-function trauma dressings and meet the hemostatic and physical protection needs in field operations, we designed a shape-adaptive bilayer hydrogel (PPTY-AP) by combining a hemostatic PPTY layer and an armor-protected AP layer to achieve complementary functions. The bottom PPTY layer, derived from a self-gelling hemostatic system and loaded with clinically validated Yunnan Baiyao herbal hemostatic powder, can closely conform to irregular wounds and undergo in situ self-gelation within 3 s upon contact with blood, efficiently activating the intrinsic coagulation pathway to achieve rapid hemostasis and promote wound repair [16,20,21,22]. The upper AP layer is an improved armor-protected hydrogel. In this work, we introduced chitosan (CS) and sodium citrate (NaCit) into the AP layer. Specifically, CS drastically shortened the UV curing time from 30 min to 50 s, addressing the drawback of excessively long photocuring time in the original protective hydrogel. Meanwhile, the corresponding irradiation dose (500 mJ/cm^2^) is far below the safe exposure threshold for skin [23], ensuring application safety. As a crystallization promoter, NaCit eliminated the inhibitory effect of the bottom layer on crystallization, enabling the upper layer to form a stable and self-renewable physical protective layer after UV curing (Figure 1).

To precisely address the critical vicious cycle of “skin trauma and bleeding—insect bites—secondary infection” in non-compressible field trauma, this study fabricated a seamlessly integrated bilayer PPTY-AP hydrogel. This layered, synergistic, and integrated design endows the PPTY-AP hydrogel with multiple functions simultaneously, including rapid hemostasis, wound repair, and physical insect-bite protection. This study not only provides a comprehensive solution to address the multiple challenges in field wound care but also demonstrates a general strategy for designing multifunctional trauma dressings.

## 2. Results and Discussion

### 2.1. Preparation of PPTY-AP Hydrogel

The PPTY-AP hydrogel is composed of a PPTY layer and an AP layer, with the structure of the hydrogel illustrated in Figure 1a. The PPTY layer is composed of tea polyphenol (TP), polyacrylic acid (PAA), polyacrylamide (PAM), and Yunnan Baiyao (YNBY), while the AP layer is composed of chitosan (CS), sodium acetate (NaAc), N,N′-Methylenebis(acrylamide) (BAM), sodium citrate (NaCit), acrylic acid (AA), acrylamide (AM) and Irgacure 2959. The chemical structural formulas of the key components that make up the PPTY-AP hydrogel are shown in Figure S1. After the PPTY layer comes into direct contact with blood, it can self-gel within 3 s to achieve rapid hemostasis. This self-gelling is driven by intermolecular hydrogen bonds and electrostatic interactions between PAA, PAM, TP and physically dispersed YNBY. YNBY activates the coagulation cascade reaction, and TP enhances tissue adhesion, thereby achieving the hemostatic effect of the PPTY layer [16]. Subsequently, the AP layer was coated on the PPTY layer and under ultraviolet irradiation (365 nm, 10 mW/cm^2^, 500 mJ/cm^2^ total dose, far below the skin safety exposure limit [3]), the AP layer underwent photocrosslinking within 50 s to form a covalent PAA-co-PAM network. This accelerated in-situ gelation and enhanced stability were driven by the introduction of CS [27,28], which formed a polyelectrolyte complex with PAA in the newly formed covalent PAA-co-PAM network. At the same time, the AP layer established interfacial hydrogen bonds and electrostatic attraction with the underlying PPTY layer to form a seamlessly integrated and stable bilayer hydrogel. In order to counteract the initial inhibitory effect of the PPTY layer on armor crystallization (Figure S2), NaCit was added to the AP network as a crystallization promoter [29], which caused NaAc and NaCit to interact ionicly with the PAA-co-PAM network, driving NaAc to crystallize on the surface and form a stable, self-renewing armor layer that protects the injured skin by providing a high-pressure physical barrier (Figure 1b and Figure S3). Compared with reported hydrogel wound dressing, PPTY-AP hydrogel presented in this work achieves an excellent balance between comfort of wearing and insect bite resistance, which is essential for delivering its three primary functions. Specifically, it exhibits a Young’s modulus close to that of skin, combined with significant resistance to puncture from insect bites (Figure 1c) [17,24,25,26].

### 2.2. Optimization and Characterization of the AP Hydrogel

**Figure 2:**
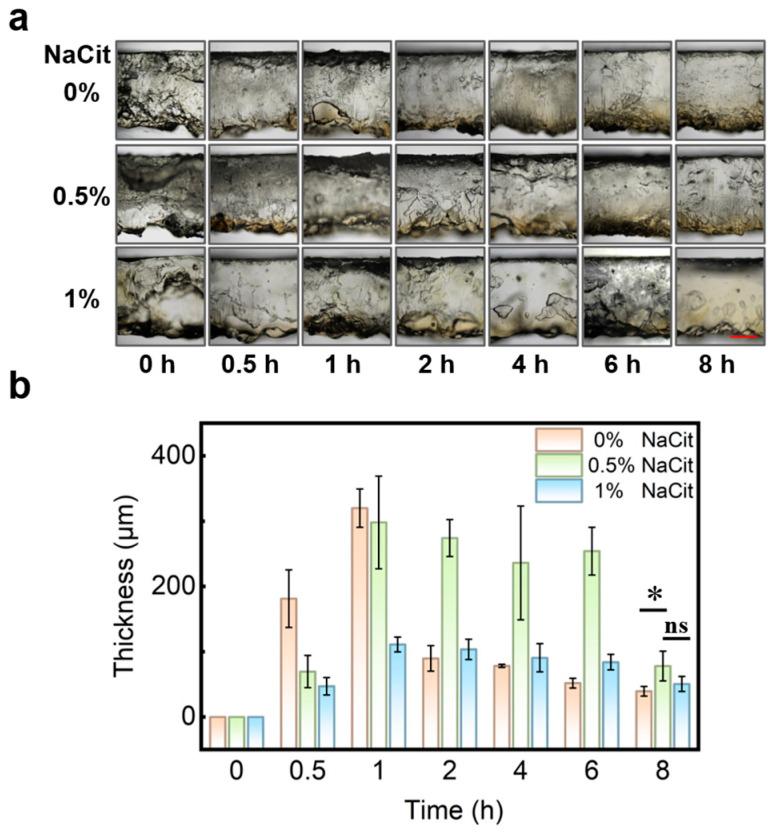
The thickness of AP armor layer with different fractions of NaCit at different times. (**a**) Photographs of AP armor layer, scale bar = 1 mm (Red line); (**b**) The thickness of armor layers, “*” represents *p* < 0.05; “ns” represents no significant difference (*p* > 0.05).

The armor layer thickness in an AP hydrogel directly influences the wound dressings’ insect-bite resistance and wearing comfort. To study the armor layer thickness, we prepared a series of AP hydrogels with varying mass fractions of NaCit. We observed a significant influence of NaCit concentration on the armor layer’s thickness using microscopy (Figure 2a). In the absence of NaCit (0%), the armor layer rapidly thickened to 320 µm within 1 h but subsequently thinned to below 100 µm after 2 h. In contrast, the armor layer in the 0.5% NaCit formulation reached 300 µm within 1 h, decreased slightly thereafter, and maintained a stable thickness of 250 µm for up to 6 h. The 1% NaCit formulation showed a slower increase to 110 µm within the first hour, followed by a gradual decline (Figure 2b). Overall, between 2 and 8 h, the 0.5% NaCit formulation produced a thicker and more stable armor layer compared to both the 0% and 1% formulations.

This effect is attributed to citrate ions (Cit^−^). Acting via the Hofmeister effect, Cit^−^ strips water from polymer hydration shells [25], promoting the precipitation of acetate ions (Ac^−^) which then combine with Na^+^ to form NaAc, ultimately leading to a thicker armor layer. This explains why the 0.5% NaCit formulation yields a thicker layer than the 0% formulation. However, in crystallization systems, Cit^−^ can also strongly bind to step sites and chelate metal ions [30]. An excess of Cit^−^ (1%) inhibits crystal nucleation through multi-dentate coordination, thereby reducing the armor layer thickness. Consequently, the 0.5% NaCit formulation in PPTY-AP hydrogels maintains a stable armor layer thickness for the longest duration, making it optimal for applications.

**Figure 3:**
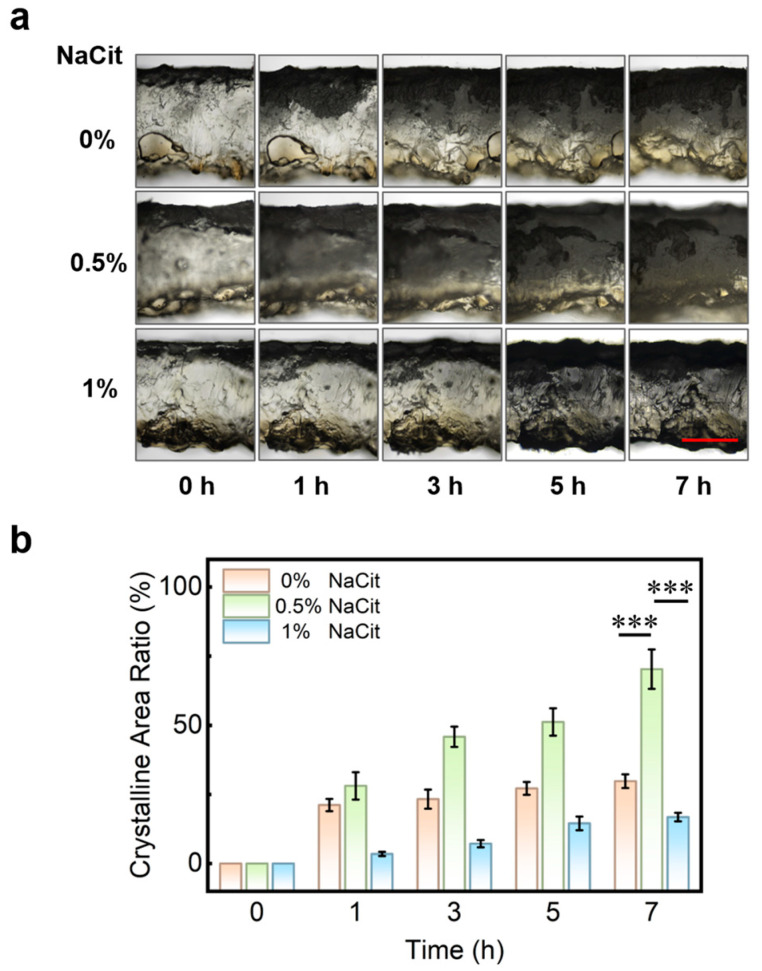
The regenerative capacity of AP armor-protective layer with different fractions of NaCit. (**a**) Photographs of AP armor layer, scale bar = 1 mm (Red line); (**b**) The newly formed crystalline area ratio of AP armor layer. “***” represents *p* < 0.001.

In field environments, where unavoidable forces like friction or sharp objects can damage the armor layer after application, its regenerative capability is a key feature for ensuring sustained protection. To evaluate the regenerative capacity, we created a fresh incision in a prepared PPTY-AP hydrogel and observed the regeneration process under a microscope. Figure 3a shows that NaCit concentration significantly affects the regeneration rate. The armor layer in the 0% NaCit formulation reformed within 1 h but slowed thereafter, covering only 30% of the damaged area after 7 h. The 0.5% NaCit formulation exhibited a fast initial regeneration rate, covering up to 70% of the area after 7 h. The 1% NaCit formulation regenerated slowly, covering merely 17% after 7 h (Figure 3b). The superior regeneration speed and coverage of the 0.5% NaCit formulation effectively mitigate the risk of hydrogel failure due to mechanical damage in use.

**Figure 4:**
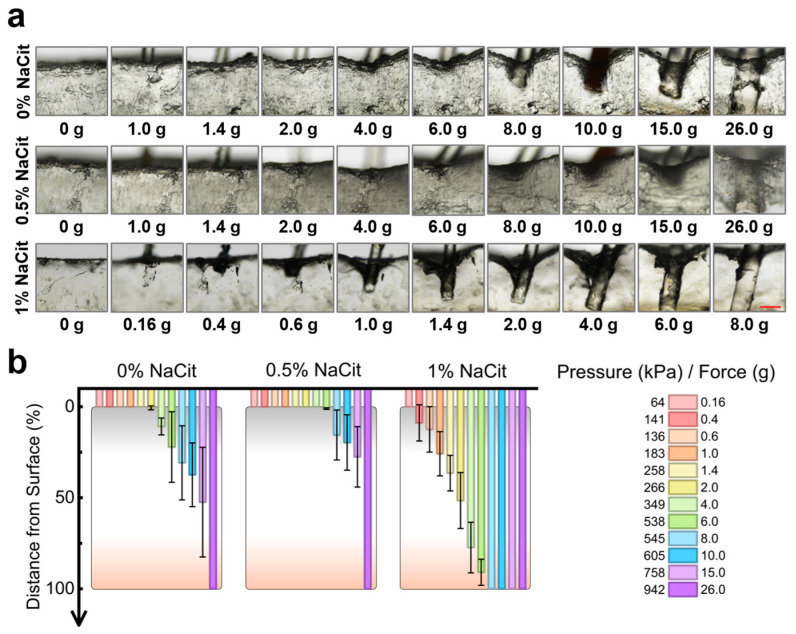
The anti-bite performance of AP armor layers. (**a**) Photographs of AP armor layers with different fractions of NaCit under different forces produced by von Frey filaments, scale bar = 1 mm (Red line); (**b**) The results of the distance from the hydrogel surface to the von Frey filament tip upon penetrating the armor layer at varying applied forces, and the relationship between the pressure generated by a von Frey filament and the applied force.

In field settings, resistance to venomous insect bites is essential. Although the absolute piercing force of blood-sucking insects (such as Aedes aegypti) is only about 18 μN, their extremely fine stylets can generate local high pressure of 12.7–637 kPa [31]. Because hydrogel puncture depends on local yield stress rather than absolute force, we used von Frey to simulate these bite mechanics [32]. By bending at specific thresholds to deliver a constant force, von Frey filaments provide a standardized, reliable method to simulate static insect bites through pressure equivalent conversion. To optimize the NaCit content for this property, we simulated insect bites using von Frey filaments. As shown in Figure 4, the 0% NaCit PPTY-AP hydrogel showed only slight deformation at 538 kPa (6.0 g force) with no penetration; significant deformation occurred at 545 kPa (8.0 g), but the layer recovered after filament withdrawal. Failure (penetration) occurred at 942 kPa (26.0 g). The 0.5% NaCit formulation began deforming at 545 kPa (8.0 g) and failed at 942 kPa (26.0 g), matching the performance of the 0% formulation at this high pressure threshold. The 1% NaCit formulation showed pronounced deformation at 183 kPa (1.0 g) and failed at 538 kPa (6.0 g). The study confirmed that a thicker armor layer withstands greater surface pressure. Given that the estimated puncture pressure of venomous insect bites ranges from 12.7 to 637 kPa [31], both the 0% and 0.5% NaCit formulations, with a failure pressure of 942 kPa, offer adequate protection. Based on the comprehensive evaluation of armor layer formation, stability, regeneration, and bite resistance, 0.5% was selected as the optimal NaCit concentration.

**Figure 5:**
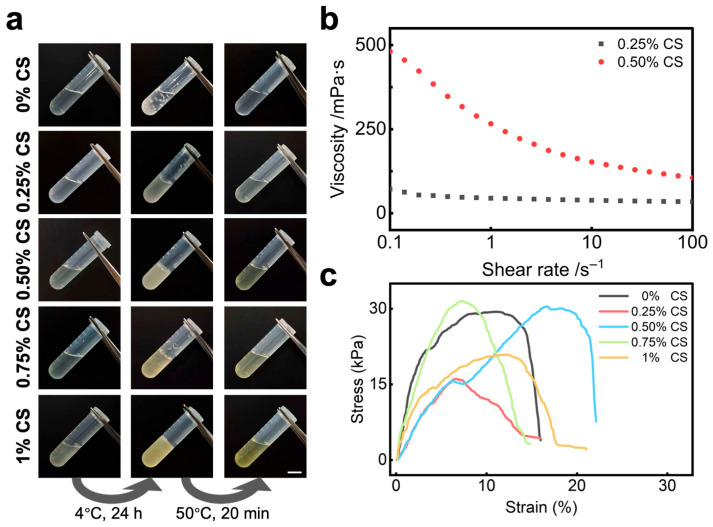
The effect of chitosan (CS) addition on the properties of AP layer hydrogels. (**a**) Photographs illustrating their freeze–thaw properties, scale bar = 1 cm; (**b**) The variation in viscosity with shear rate; (**c**) Stress–strain curves of AP hydrogels with different fractions of CS.

Chitosan (CS) incorporation enhances the AP-layer hydrogel system by improving the stability of the precursor and the crosslinked hydrogel (Figure S4), boosting conformability to skin deformation, and providing an application-friendly viscosity for greater handling convenience. In addition, CS accelerate the photopolymerization process by promoting uniform dispersion of monomers, scavenging oxygen free radicals and forming polyelectrolyte complexes [33,34]. To further optimize PPTY-AP hydrogel performance, we prepared a series of precursor solutions with different CS mass fractions (0%, 0.25%, 0.5%, 0.75%, 1%). First, we investigated the storability of the AP hydrogel precursor solutions. The solution with 0% CS gelled after 24 h at 4 °C and formed flocculent precipitates upon heating at 50 °C for 20 min, rendering it ineffective. Solutions with 0.25%, 0.5%, and 0.75% CS also gelled at 4 °C but reverted to a clear, transparent liquid identical to the freshly prepared solution after the 50 °C heat treatment. The 1% CS solution gelled at 4 °C and formed flocculent precipitates upon heating (Figure 5a). This outcome results from changes in the PAA-CS stoichiometric ratio with varying CS concentration, which alters the non-covalent interactions and phase behavior [35,36]. Without CS (0%), strong salting-out by NaCit dehydrates and aggregates PAA, causing precipitation at both 4 °C and 50 °C [37]. At intermediate CS concentrations (0.25–0.75%), PAA excess forms soluble polyelectrolyte complexes; reversible physical gelation occurs at 4 °C via non-covalent bonds, which are disrupted at 50 °C to restore the liquid state [38,39]. At 1% CS, the solution approaches charge stoichiometry, forming electrically neutral, hydrophobic aggregates that irreversibly solidify at 50 °C via amidation or enhanced hydrophobic interactions [40,41]. For practical use, formulations with 0.25–0.75% CS are suitable.

To evaluate the spreadability of the hydrogel during application, we assessed the viscosity of the precursor solutions via rheological tests. As shown in Figure 5b, the solution with 0.25% CS exhibited low, shear-rate-independent viscosity, leading to high fluidity and easy diffusion upon application. The solution with 0.5% CS displayed pronounced shear-thinning behavior, its viscosity decreased with increasing shear rate, and a higher initial viscosity, which is crucial for controlled application and adherence [5]. The 0.75% CS solution was too viscous to form a uniform layer on the testing stage, preventing reliable measurement. Viscosity increased and fluidity decreased with higher CS concentrations. The 0.5% CS formulation demonstrated the most favorable rheological properties for spreading.

To evaluate the skin-conformability of the AP-layer hydrogel, we conducted the tensile testing. From Figure 5c, the tensile strength for AP layers with 0%, 0.5%, and 0.75% CS was approximately 30 kPa; it was about 15 kPa for 0.25% CS and 20 kPa for 1% CS. More importantly, the 0.5% CS formulation achieved the highest fracture strain of ~20%, compared to ~15% for the others. This performance is attributed to network structure: the 0% CS hydrogel forms a physical-covalent network via the PAM-PAA covalent network and hydrogen bonds from freeze–thaw cycles, yielding good strength and strain [42,43]. At 0.25% CS, sparse CS chains fail to form a reinforcing network and disrupt main-chain physical crosslinking. At 0.75% and 1% CS, excessive ionic crosslinking or CS self-aggregation under high salinity restricts chain mobility, increasing brittleness [44,45,46,47]. The 0.5% CS formulation optimizes the dual-network structure, where sacrificial bonds from PAA-CS and CS-citrate interactions efficiently dissipate energy, leading to peak performance [48,49,50]. Based on the comprehensive assessment of precursor solution storability, spreadability, and hydrogel mechanical properties, 0.5% CS was identified as the optimal concentration for the AP hydrogel precursor solution.

### 2.3. Characterization of the PPTY-AP Hydrogel

**Figure 6:**
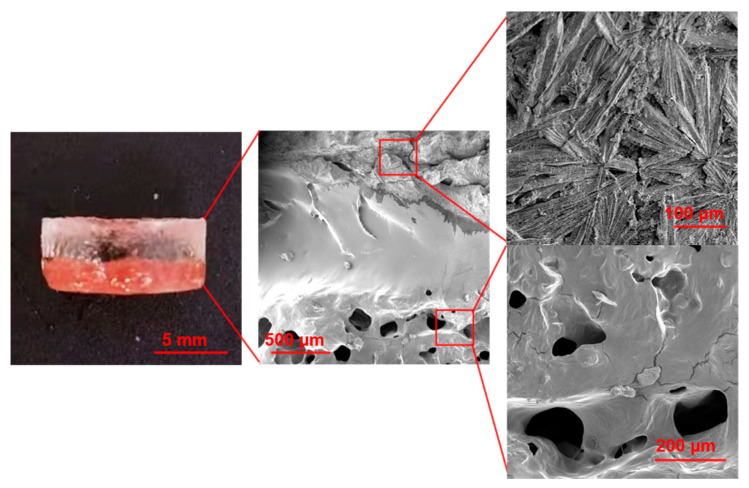
The scanning electron microscopy images of cross-sectional morphological analysis of the double-layer hydrogel.

Following the optimization of NaCit and CS concentrations, all subsequent experiments were conducted using the identified optimal formulation. To study the morphology of the PPTY-AP hydrogel and assess the interfacial bonding between the PPTY and AP layers, we analyzed its cross-sectional structure using scanning electron microscopy (SEM). As shown in Figure 6, the hydrogel exhibits a distinct bilayer structure. The upper surface appears wrinkled with visible NaAc crystals, corresponding to the armor layer of the AP component designed to prevent insect bites. The deeper region of this upper layer is densely packed, representing the non-crystalline part of the AP layer. The lower layer displays a porous structure, characteristic of the PPTY layer, which facilitates rapid blood absorption and subsequent hemostasis. Notably, a seamless interface is observed between the two layers, indicating strong interfacial adhesion.

**Figure 7:**
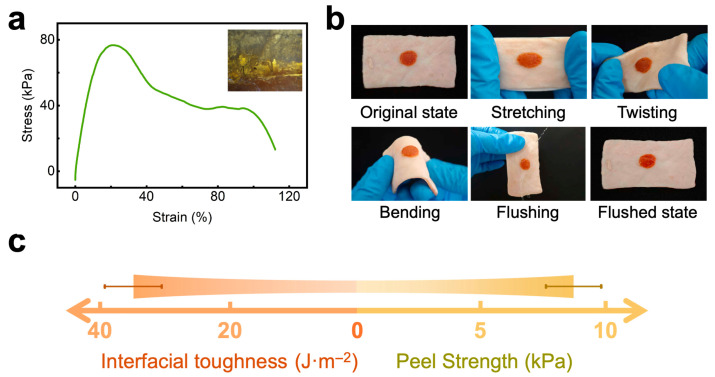
Tensile properties and tissue adhesion properties of PPTY-AP hydrogel. (**a**) The stress–strain curve of hydrogel and the interface image of the two hydrogel layers after the tensile test (insert image). (**b**) Tissue adhesion test results of the hydrogel on rabbit skin. (**c**) 180° peel strength and interfacial toughness of the hydrogel.

Then, we evaluated the skin conformability of the PPTY-AP hydrogel via tensile testing. The results show that the bilayer hydrogel can withstand a maximum tensile strength of 75 kPa, at which point the upper (AP) layer fractures, corresponding to a strain of 25% (Figure 7a). The lower layer (PPTY hydrogel) demonstrates a significantly higher fracture strain of up to 100%. During tensile deformation, the bilayer acts synergistically rather than as separate layers. Its excellent mechanical properties come from strong interfacial bonding between the rigid AP and flexible PPTY layers, formed by hydrogen bonds and polyelectrolyte complexation. This stable interface avoids delamination, enables effective stress transfer, and lets the elastic PPTY layer support and dissipate stress for the stiffer AP layer under tension. Compared to the AP-layer hydrogel alone, the PPTY-AP hydrogel exhibits a slightly increased fracture strain and a 2.5-fold greater tensile strength. The resulting hydrogel modulus matches that of human dermis [51]. Based on the stress–strain curve during the elastic deformation stage, we determined that the Young’s modulus of the PPTY-AP hydrogel is 0.824 MPa, matching that of adult human skin (0.850 MPa) [52]. Microscopic examination of the fractured surface after tensile testing confirmed that the two layers remained tightly bonded without delamination (Figure 7a), indicating that the double-layer structure plays a key role in balancing strength and flexibility during tensile deformation.

In addition to tensile testing, we also evaluated the compressive properties of the PPTY-AP hydrogel to determine its ability to withstand external environmental loads. To assess the compressive behavior of the soft hydrogel and avoid boundary artifacts and clamp slippage common in standard universal testing machines, the static gravity compression method was used [53]. As shown in Figure S5, the PPTY-AP hydrogel can withstand pressures up to 186 kPa with a deformation of approximately 35% at this pressure; with 100 kPa as the minimum mechanical support threshold, this hydrogel meets the required mechanical support requirements [54]. Within the 0–8% strain range, its compressive modulus is 0.146 MPa, falling within the soft tissue fit range of 0.01–1 MPa, and its mechanical properties meet soft tissue matching requirements [54]. This excellent mechanoelasticity primarily stems from the structural asymmetry introduced by the upper AP layer. The rapid photocrosslinking of the CS network and the high-density crystallization of NaAc on the surface together construct a rigid microstructural barrier [19]. Under vertical compression, this surface layer effectively disperses stress over a larger load-bearing area, preventing localized mechanical failure while protecting the underlying flexible PPTY layer from compression damage. This compressive strength is crucial in applications: even under complex environments, the hydrogel maintains its structural volume and the integrity of its internal porous network. Preservation of the pore structure is essential for continuous exudate absorption, accumulation of clotting factors, and maintaining the moist microenvironment necessary for wound healing.

Tissue adhesion is crucial for the PPTY-AP hydrogel to achieve rapid hemostasis and promote wound healing [16]. We first assessed this property using a 180° peel test on the rabbit skin. As demonstrated in Figure 7b, the hydrogel remained firmly adhered without detachment or gap formation when the skin was subjected to stretching, twisting, bending, or water rinsing. The measured peel strength was 8.7 kPa, with an interfacial toughness of approximately 35 J·m^−2^ (Figure 7c). This toughness value is about 3.5 times higher than that of clinically used dressings and bandages (~10 J·m^−2^) [55], showing the strong tissue adhesion of the PPTY-AP hydrogel.

The excellent tissue adhesion and mechanical properties of PPTY-AP hydrogel show the importance of its bilayer structure for tissue adaptability, which helps biomaterials match tissue movement and prevent stress shielding, micromotion, and chronic inflammation [54]. Traditional homogeneous hydrogels have an inherent conflict: flexible hydrophilic gels fit tissues well but lack strength, while stiff ones show poor adhesion. Layered structures provide an effective solution to this dilemma; for example, Joshi et al. developed a 4D-printed deformable alginate/methylcellulose dual-network hydrogel that achieves anisotropic self-rolling via interlayer swelling differences, enabling sutureless nerve encapsulation, reducing complications, and promoting nerve regeneration [56]. Inspired by this, the PPTY-AP bilayer hydrogel uses a decoupled design: its bottom PPTY layer gels within 3 s to conform to irregular tissues, and the top AP layer serves as a mechanical barrier. By mimicking the gradient of natural tissues, this design separates interfacial biocompatibility from external mechanical stability, ensuring the material remains adaptable and structurally intact under physiological stress.

Wound infection significantly impedes healing, making antibacterial activity a desirable property for an ideal wound dressing [57,58]. To explore this capability, we placed the hydrogel in the center of culture media inoculated with *Escherichia coli* (*E. coli*) and *Staphylococcus aureus* (*S. aureus*). After 24 h of incubation, the hydrogel exhibited antibacterial activity against both strains, with a more pronounced effect against *S. aureus*. The inhibition zone area of *E. coli* and *S. aureus* were 2.47 ± 0.07 cm^2^ and 4.66 ± 0.09 cm^2^ (Figure S6).

The in vitro cytocompatibility and hemocompatibility of the bottom PPTY hydrogel layer, which is in direct contact with the skin and wound tissue, have been systematically verified [16]. The upper AP armor-protective layer of the PPTY-AP bilayer hydrogel does not make direct contact with the skin or wound surface, and the raw materials used in the AP layer are widely used in wound dressing. Therefore, the material shows good biocompatibility and satisfies the safety requirements for wound dressings.

**Figure 8:**
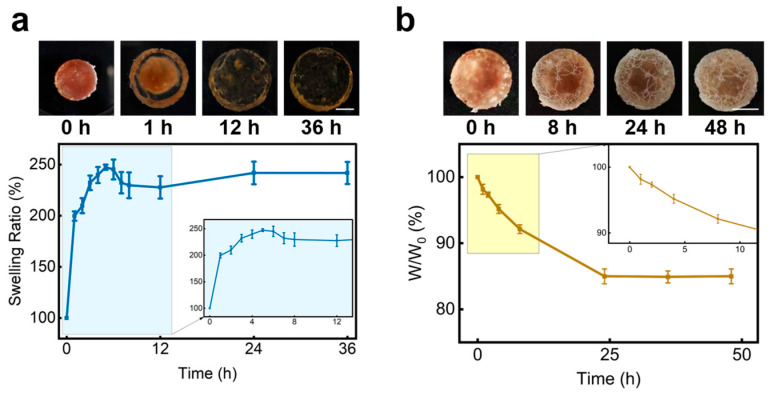
The swelling ratio and water retention property of PPTY-AP hydrogel. (**a**) Typical images and swelling ratio curve of PPTY-AP hydrogel, scale bar = 5 mm. (**b**) Typical images and water retention curves of PPTY-AP hydrogel, scale bar = 5 mm.

The swelling ratio of a hydrogel is related to its capacity for rapid fluid absorption, which is essential for hemostasis [16]. As shown in Figure 8a, the PPTY-AP hydrogel swelled rapidly within the first 5 h. The swelling speed then decreased until reaching an inflection point at 12 h, after which it slowly increased again and stabilized after 24 h. This profile can be explained as follows: initially, both layers absorb water, causing the rapid increase. Subsequently, the degradation of the PPTY layer leads to a decline in the overall swelling ratio, with complete PPTY degradation occurring by 12 h. Thereafter, the continued swelling of the stable AP layer dominates, reaching equilibrium by 24 h. For practical field wound management, the initial rapid swelling facilitates hemostasis. Furthermore, the degradation of the PPTY layer after 12 h is advantageous for subsequent wound redressing and further medical care. Water retention is crucial for the environmental and structural stability of hydrogels, as water helps maintain the morphology, flexibility, and functionality of the polymer network [59,60,61]. Furthermore, hydrated ions can help stabilize the system by reducing water evaporation through the formation of a hydrated shell [62,63,64]. As shown in Figure 8b, the PPTY-AP hydrogel maintained its shape and integrity after 48 h at 25 °C and 40% RH, while retaining approximately 85% of its initial weight, indicating that the hydrogel network remained structurally stable under ambient exposure. This excellent water retention is attributed to the high concentration of NaAc within the hydrogel network, where water molecules form hydration shells around the ions, hindering evaporation [64,65]. Compared to commercial dressings, this performance is superior to traditional gauze (>90% water loss) and high-alginate hydrogels (~60% water loss), approaching the retention level of synthetic polymer hydrogels. The dynamic self-sealing barrier of this PPTY-AP hydrogel also offers an advantage over static occlusive dressings [66,67,68]. The superior water retention of the PPTY-AP hydrogel is therefore highly beneficial for promoting wound healing.

### 2.4. In Vivo Hemostatic Performance of the PPTY-AP Hydrogel

**Figure 9:**
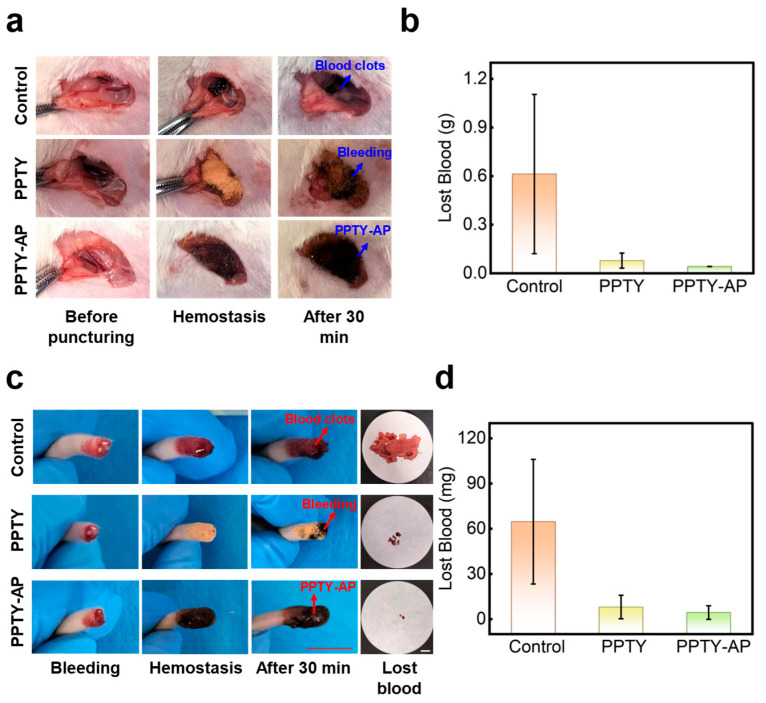
In vivo hemostatic performance of PPTY-AP hydrogel. (**a**) Photographs of the femoral artery hemostasis process. (**b**) Quantitative representation of femoral artery blood loss. (**c**) Photographs of the tail vein hemostasis process. (**d**) Quantitative representation of tail vein blood loss, scale bar = 1 cm.

To evaluate the in vivo hemostatic capability of the PPTY-AP hydrogel, we developed rat femoral artery and tail vein bleeding models. In the femoral artery model, the control group (which received a puncture without hemostasis intervention) exhibited a blood loss of 0.61 ± 0.49 g, with bleeding ceasing spontaneously after 30 min. Application of the PPTY-layer hydrogel powder (PPTY group) significantly reduced blood loss to 0.08 ± 0.05 g; however, minor oozing was observed 10 min post-hemostasis. Application of the PPTY-AP bilayer hydrogel (PPTY-AP group) further reduced the blood loss to 0.04 ± 0.0007 g, shortened the hemostasis time to 1 min, and prevented any subsequent oozing over the following 30 min (Figure 9a,b). A similar trend was observed in the tail vein bleeding model. The blood loss for the control group, PPTY group, and PPTY-AP groups were 64.67 ± 41.36 mg, 8.00 ± 7.81 mg, and 4.33 ± 4.51 mg, respectively (Figure 9c,d). These results demonstrate the superior in vivo hemostatic efficacy and applicability to irregular anatomical sites of the PPTY-AP hydrogel compared to PPTY powder alone. The porous PPTY powder exhibits strong adhesion to wet tissue and can activate the endogenous clotting pathway. It rapidly absorbs blood, undergoes self-gelation, and firmly anchors to the wound surface for hemostasis, while concentrating platelets and coagulation factors to accelerate blood clotting [16]. However, during active bleeding, the flowing blood can wash away the PPTY powder during its pre-gelation state, resulting in an insufficient quantity, which then leads to oozing. The incorporation of the AP layer in the PPTY-AP hydrogel provides an effective physical barrier that mitigates this oozing.

To further verify the clinical translational value of this study, we quantitatively compared the PPTY-AP hydrogel with commercial hemostatic agents in consistent rodent models. Commercial chitosan-based hemostats (Celox) typically require 2–5 min to achieve effective hemostasis, with blood loss ranging from 108 to 538 mg in the tail vein bleeding model [69]. Kaolin-based hemostatic dressings (QuikClot Combat Gauze) take 3–18 min to stop bleeding and form unstable clots prone to recurrent oozing [70,71]. In contrast, the PPTY-AP hydrogel achieves rapid hemostasis within 1 min, reduces blood loss by one order of magnitude, and does not require prolonged manual compression. This design overcomes the critical drawbacks of conventional commercial hemostats, including unstable clots, secondary micro-seepage, and dressing displacement. In summary, the PPTY-AP bilayer hydrogel can control incompressible traumatic hemorrhage more quickly and reliably.

### 2.5. Wound Healing Promotion by the PPTY-AP Hydrogel

**Figure 10:**
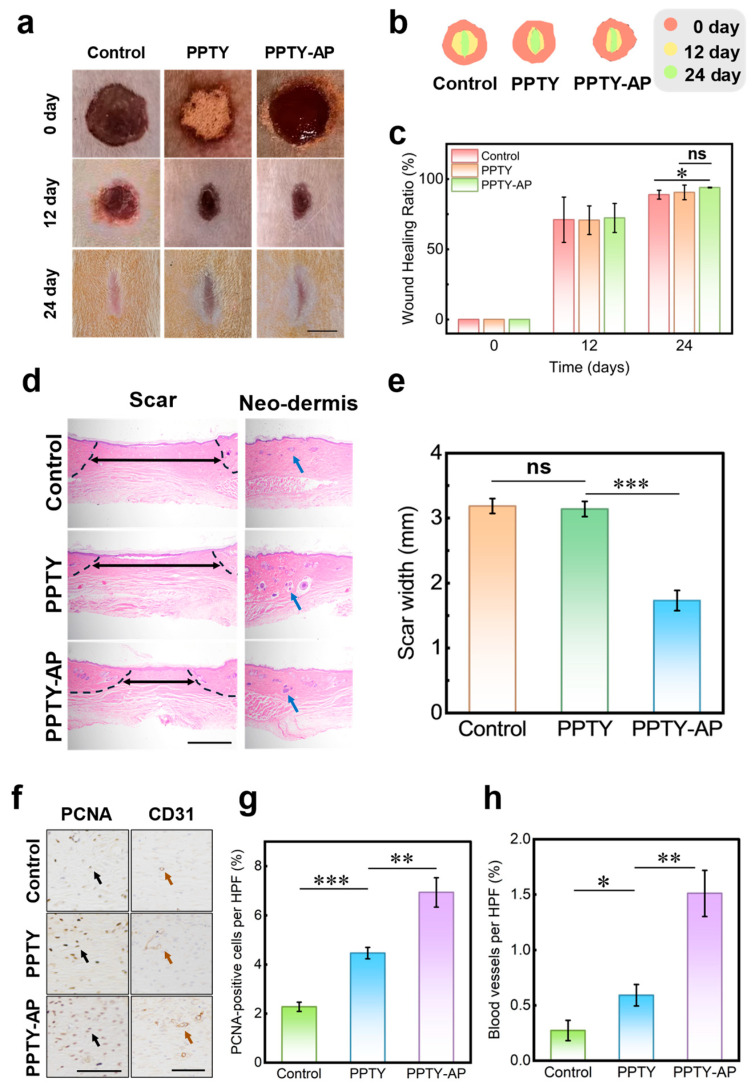
Results of in vivo wound healing study. (**a**) Photographs illustrating wound progression in each group over 24 days of treatment, scale bar = 5 mm. (**b**) Color-coded wound maps for each group over 24 days. (**c**) Quantitative representation of wound healing in each group over 24 days. (**d**) H&E staining images of wound tissue in each group in 24 days. Black dashed lines show the outline of the neo-dermis regeneration and granulation tissue, black double-headed arrows indicate the width of scar, and blue arrows indicate the hair follicle, scale bar = 1 mm. (**e**) Quantitative representation of scar width in each group of wounds in 24 days. (**f**) Representative images of Immunohistochemical staining for proliferating cell nuclear antigen (PCNA) and the angiogenesis marker CD31. Black arrows indicate PCNA-positive cell. Brown arrows indicate blood vessel, scale bar = 100 μm. (**g**) Quantification of PCNA-positive cells from images acquired at high power field (HPF). (**h**) Quantification of CD31-positive capillaries from images acquired at HPF. “*” represents *p* < 0.05; “**” represents *p* < 0.01; “***” represents *p* < 0.001; “ns” represents no significant difference (*p* > 0.05).

The PPTY-AP hydrogel also functions as a wound dressing to promote skin wound healing. To verify this, circular wounds on rat dorsum were treated with PBS (control group), 100 mg of PPTY powder (PPTY group), or the PPTY-AP hydrogel (PPTY-AP group). As shown in Figure 10a–c, the wounds treated with either PPTY powder or the PPTY-AP hydrogel showed better healing outcomes compared to the control group by day 12. By day 24, wounds in all groups were nearly closed, with the PPTY-AP hydrogel group exhibiting the smallest residual scar. Histological analyzation via Hematoxylin and Eosin (H&E) staining revealed that the PPTY-AP hydrogel-treated group had the smallest scar width (indicated by black double-headed arrows). Furthermore, both the PPTY powder and PPTY-AP hydrogel groups showed a greater number of hair follicles (blue arrows) in the neodermis compared to the control (Figure 10d,e). To study re-epithelialization of wound, immunohistochemical staining for Proliferating Cell Nuclear Antigen (PCNA), a marker for G1/S phase proliferation, was performed. The proportion of PCNA-positive cells (black arrows) was significantly higher in the PPTY-AP hydrogel group than in the control and PPTY groups (Figure 10f,g). Neovascularization within the granulation tissue was evaluated by staining for CD31, a marker for endothelial cells and angiogenesis. The density of CD31-positive capillaries (brown arrows) was significantly higher in the PPTY-AP hydrogel group compared to the other two groups (Figure 10f,h). The PPTY hydrogel promotes wound healing superior to the control by providing a moist healing environment through strong adhesion, coupled with the anti-inflammatory, pro-proliferative, and pro-angiogenic effects of its components (TP and YNBY) [16]. The PPTY-AP hydrogel combines these advantages of the underlying PPTY layer with the stable physical barrier of the AP layer. Additionally, CS in the formulation further stimulates fibroblast function, modulates collagen deposition, and inhibits scar fibroblast proliferation and excessive collagen secretion, thereby reducing scar formation [72,73]. Collectively, the PPTY-AP hydrogel effectively promotes the healing of full-thickness skin wounds.

Traditional commercial wound dressings such as Tegaderm only provide passive moisturization, leading to slow healing [74]. By contrast, the PPTY-AP bilayer hydrogel promotes cell proliferation and angiogenesis through the synergistic effects of TP, YNBY and CS. It achieves 72% wound closure by day 12 and nearly full closure (94%) by day 24, with better tissue remodeling and less scarring. Instead of just covering wounds, it supports high-quality full-thickness regeneration, showing clinical advantages over traditional dressings.

## 3. Conclusions

In this study, we developed a shape-adaptive bilayer PPTY-AP hydrogel for field trauma management, integrating functions of rapid hemostasis, wound healing and physical protection. The AP layer precursor solution forms a hydrogel with a 250–300 µm thick armor layer after 50 s of UV curing, enabling in situ formation on wounded skin that acts as a structural shield. In vivo hemostasis experiments demonstrated that the underlying PPTY powder gels instantly upon blood contact within 3 s, exerting a rapid hemostatic effect. In a full-thickness skin defect model, the PPTY-AP group exhibited the fastest wound healing rate, with 94% wound closure on day 24, along with the smallest residual scar width. This performance was superior to that of the standard commercial wound dressing Tegaderm. The PPTY-AP bilayer hydrogel, with its capabilities for rapid in situ formation, efficient hemostasis, and insect bite prevention, not only provides a preliminary solution to the “traumatic bleeding-insect bite” challenge in field environments but also offers a design strategy for next-generation multifunctional wound dressings for field trauma first-aid.

## 4. Materials and Methods

### 4.1. Materials

Poly(acrylic acid) solution (PAA, 50 wt%), polyacrylamide (PAM, Mw: 2,000,000–14,000,000), tea polyphenol (TP, 98%), sodium acetate (NaAc, purity > 99%), sodium citrate (NaCit, purity > 99%), chitosan (CS, deacetylated ≥ 95%, viscosity 100–200 mPa.s from shrimp shells), N,N′-Methylenebis(acrylamide) (BAM, purity > 99%), PBS buffer solution, and paraformaldehyde were purchased from Aladdin Co., Ltd. (Shanghai, China). Acrylamide (AM, purity > 99%), acrylic acid (AA, purity > 99%), and acetic acid were purchased from Macklin Biochemical Technology Co., Ltd. (Shanghai, China). Yunnan Baiyao (YNBY, powder) was purchased from Yunnan Baiyao Group Co., Ltd. (Yunnan, China). Irgacure 2959 was purchased from IGM Resins (Shanghai) Management Co., Ltd. (Shanghai, China).

### 4.2. Fabrication of PPTY-AP Hydrogel

#### 4.2.1. Preparation of PPTY Powder

1.23 g TP and 5 mg standardized commercial YNBY powder were dissolved in 5 mL of a 10 wt% PAA solution. Subsequently, PAM powder was added to form 8 wt% solution, which was then allowed to swell at 37 °C for 100 min. Finally, the resulting hydrogel was freeze-dried and thoroughly ground to obtain PPTY powder.

#### 4.2.2. Preparation of Armor-Protected Hydrogel Precursor Solution

CS powder was dissolved in a 0.26 M acetic acid solution and stirred until completely dissolved to prepare a 5% *w*/*v* CS solution. AM and AA were dissolved in deionized water at 60 °C, and different mass fractions of NaCit (0 wt%, 0.5 wt%, 1 wt%) were respectively dissolved in the aforementioned solution. After complete dissolution, NaAc, different mass fractions of the 5% *w*/*v* CS solution (0 wt%, 0.25 wt%, 0.5 wt%, 0.75 wt%, 1 wt%), BAM, and Irgacure 2959 were added to the solution. The mixture was stirred until fully dissolved and then sonicated to remove air bubbles, yielding the hydrogel precursor solution. The solution was stored in a refrigerator at 4 °C for further use. Table 1 shows the compositions of AP hydrogel precursor solutions with varying mass fractions of NaCit and CS.

#### 4.2.3. In Situ Formation of PPTY-AP Hydrogel

An excessive amount of PPTY powder was applied onto the wound surface. After all the exudate had been absorbed and the PPTY powder had reached a semi-gel state, the AP hydrogel precursor solution, which had been pre-equilibrated to room temperature, was dripped onto the PPTY layer. Subsequently, the system was exposed to UV light (365 nm, 10 mW/cm^2^) for 50 s to induce gelation.

### 4.3. Characterization of the AP Layer of PPTY-AP Hydrogel

PPTY-AP hydrogel samples with the size of 20 mm × 10 mm × 5 mm and different ratio of NaCit (0%, 0.5% and 1%), were prepared, and sectioned into 2 mm-thick specimens using a scalpel. The generated AP layer of sectioned hydrogel samples were observed under an inverted microscope (Nikon Corporation, Tokyo, Japan), and recorded at different time intervals. After 1 h, new incisions were made on these hydrogel samples. The thickness and coverage area ratio of the newly formed crystals on the hydrogel surface were recorded at different time to assess the regeneration capacity of the AP layer.

To evaluate the mechanical bite resistance of the AP layer, von Frey filaments (Ugo Basile, Gemonio, Italy) with different forces were applied to vertically puncture the hydrogels. To simulate the local stress concentration of insect bites based on the size scaling principle, the absolute buckling force was converted into contact pressure (P) by dividing the buckling force (F) of each monofilament by the cross-sectional area (A) of a particular monofilament tip. The corresponding pressure generated by the filaments on the hydrogel, in relation to the applied force, is listed in Table 2. Hydrogel samples for the puncture test were prepared following the same procedure as for the AP layer thickness test. 1 h after gelation, von Frey filaments with different forces were applied to vertically puncture the crystalline surface of hydrogels. The force and pressure required to rupture the PPTY-AP hydrogel AP layer, as well as the puncture depth, were observed and recorded under the microscope.

### 4.4. PPTY-AP Hydrogel Characterization

#### 4.4.1. Freeze–Thaw Stability

The prepared PPTY-AP hydrogel precursor solution was stored in a refrigerator at 4 °C. After 24 h, the solution was taken out and heated in a 50 °C water bath for 20 min. The morphology of the solution was then observed.

#### 4.4.2. Rheological Properties

The viscosity of the AP layer hydrogel precursor solution was evaluated using a Discovery HR-20 hybrid rheometer (TA Instruments, New Castle, DE, USA). The measurement was conducted at 25 °C in rotational mode, with the shear rate set from 0.1 to 100 s^−1^.

#### 4.4.3. Tensile Properties of PPTY-AP Hydrogel

PPTY-AP hydrogel samples with the size of 20 mm × 10 mm × 5 mm were prepared. The tensile properties of the hydrogel samples were tested using an HKE-3511 peel strength tester (Kexing Precision Instruments Co., Ltd., Shenzhen, China) at 25 °C with a crosshead speed of 1 mm/min. The interfacial state between the two layers of the hydrogel after the tensile test was observed under a microscope.

#### 4.4.4. The Morphology of PPTY-AP Hydrogel

The morphology of the prepared hydrogels was analyzed using a field emission scanning electron microscope (FE-SEM, Thermo Fisher Scientific Inc., Waltham, MA, USA). The freeze-dried hydrogels were placed on sample stages, sputter-coated with gold, and subsequently observed under the SEM.

#### 4.4.5. Compression Properties of PPTY-AP Hydrogel

Cylindrical PPTY-AP hydrogel samples (10 mm in diameter, 10 mm in height) were prepared. The samples were placed at the center of two parallel rigid plates, with the upper plate being a lightweight acrylic plate to ensure uniform stress distribution. Compressive stress was generated by sequentially placing standardized calibration weights (100 g, 200 g, 500 g, 1500 g) at the center of the upper plate. After each increase in load, a 60 s equilibrium period was set to ensure that the hydrogel network reached steady-state deformation. A camera was placed perpendicular to the sample axis to capture deformation images at each equilibrium state. The images were then processed using ImageJ (1.54 g) to measure the change in sample height, obtaining the hydrogel compressive stress–strain relationship. The compressive modulus was determined by the slope of the linear elastic region of the obtained compressive stress–strain relationship (evaluated within the 0–8% strain range).

#### 4.4.6. Tissue Adhesiveness of PPTY-AP Hydrogel

At 25 °C, cylindrical PPTY-AP hydrogel specimens (diameter = 10 mm, height = 5 mm) were prepared on rabbit skin. The adhesiveness of the specimens was observed while the rabbit skin was subjected to stretching, twisting, bending, and water rinsing. Additionally, rectangular PPTY-AP hydrogel samples (50 mm × 10 mm × 5 mm) were prepared, and adhered to the surface of ex vivo rabbit skin. A pressure of 5 kPa was applied for 20 s, after which the 180° peel strength of the hydrogel was tested using a peel strength tester to calculate the interfacial toughness. Each sample was peeled at 180° and a speed of 1 mm/min at 25 °C. The interfacial toughness was calculated using the following formula:

(1) $$Interficial\;Toughness=\frac{2\times F_{m}}{W}$$

where *F_m_* is the average peel force, and *W* is the width of the hydrogel adhesion area.

#### 4.4.7. Swelling Ratio and Water Retention Capacity

To investigate the swelling ratio of the prepared hydrogels, cylindrical hydrogels (diameter = 10 mm, height = 5 mm) were accurately weighed and immersed in PBS buffer at 37 °C. The hydrogels were taken out at predetermined time intervals, the surface water was gently removed with filter paper, and the weight change was recorded. The swelling ratio was calculated using the following formula:

(2) $$Swelling\;Ratio=\frac{W_{t}}{W_{0}}\times 100\%$$

where *W*_0_ is the initial weight of the hydrogel, and *W_t_* is the weight of the hydrogel after absorbing water at each time interval.

To evaluate the water retention capacity, cylindrical hydrogels (diameter = 10 mm, height = 5 mm) were prepared, accurately weighed, and placed at 25 °C and 40% relative humidity (RH). The hydrogels were weighed at predetermined time intervals, and the weight change was recorded. The water retention ratio was calculated using the following formula:

(3) $$Water\;Retention\;Ratio=\frac{W_{t}}{W_{0}}\times 100\%$$

where *W*_0_ is the initial weight of the hydrogel, and *W_t_* is the weight of the hydrogel at each time interval.

#### 4.4.8. Antibacterial Properties

The antibacterial properties of the hydrogel samples were assessed by determining the inhibition zones formed against *Escherichia coli* and *Staphylococcus aureus*. A total of 1 mL of a bacterial suspension [1 × 10^6^ colony-forming units (CFU)/mL] was inoculated onto a Mueller-Hinton (MH) plate with nutrient agar medium (Guangzhou, China). The hydrogel samples (cylinders with d = 10 mm, h = 5 mm) were then placed in the center of the plates. After incubating at 37 °C for 24 h, the area of the inhibition zones were recorded to evaluate the antibacterial effect.

### 4.5. In Vivo Hemostatic Performance Evaluation

The in vivo hemostatic performance of the PPTY-AP hydrogel was evaluated using two wound models in rats: femoral artery puncture and tail vein transection. Sprague-Dawley (SD) male rats (6 weeks old, weighing 200 ± 20 g) were purchased from Guangxi Medical University. All animal studies were conducted in accordance with the guidelines approved by the Institutional Animal Care Committee of Guangxi University (NO. GXU-2024-207). The rats were randomly divided into three groups: a control group, a PPTY monolayer hydrogel group, and a PPTY-AP bilayer hydrogel group. Rats were anesthetized with chloral hydrate (7% *w*/*v*, 0.7 mL per 100 g body weight). In the femoral artery puncture model, the femoral artery was isolated and then punctured using a 25 G needle. For the control group, spontaneous hemostasis was allowed to occur. In the PPTY monolayer hydrogel group, hemostasis was achieved by applying 100 mg of PPTY powder. In the PPTY-AP bilayer hydrogel group, 100 mg of PPTY powder was first applied for hemostasis, followed by coating with the AP hydrogel precursor solution to form the PPTY-AP hydrogel. Pre-weighed cotton was used to absorb the exuded blood, and the weight of the blood-soaked cotton was recorded after bleeding ceased. In the tail vein transection model, a pre-weighed clean filter paper was placed beneath the rat’s tail, and then approximately 2 cm of the tail tip was amputated. The hemostatic procedures for the three groups were identical to those in the femoral artery puncture model, and the weight of the blood-soaked filter paper was recorded after hemostasis.

### 4.6. In Vivo Wound Healing Study

Full-thickness circular skin wound with a diameter of 10 mm were created on the dorsal surface of male Sprague-Dawley (SD) rats (6 weeks old, weighing 200 ± 20 g). Rats were randomly divided into three groups: a control group, a PPTY monolayer hydrogel group, and a PPTY-AP bilayer hydrogel group, with 6 rats in each group. Sterile PBS was applied to the wounds in the control group. The wounds in the PPTY monolayer hydrogel group were covered with 100 mg PPTY powder, while those in the PPTY-AP bilayer hydrogel group were covered with PPTY-AP hydrogel. To evaluate the healing process, wounds were photographed on post-treatment days 12 and 24. The wound healing ratio was calculated as follows:

(4) $$Wound\;Healing\;Ratio=\frac{\left(A-A_{t}\right)}{A}\times 100\%$$

where *A* is the initial wound area and *A_t_* is the wound area at a designated time point. After 24 days, the rats were sacrificed, and tissue from the wound site was collected. The tissue was fixed in 4% formalin solution for 24 h, dehydrated, and embedded in paraffin. The paraffin-embedded samples were sectioned into 5 μm slices. The sections were deparaffinized with xylene and dehydrated using a graded alcohol series. Subsequently, they were stained with hematoxylin and eosin (H&E) for histological analysis under a microscope to assess wound healing. Immunohistochemistry was performed to detect the cell proliferation marker (proliferating cell nuclear antigen, PCNA) and to evaluate neovascularization marker (CD31). The dehydrated sections mentioned above underwent antigen retrieval using citrate buffer (10 mmol/L, pH 6.0). Endogenous peroxidase activity was quenched with 3% hydrogen peroxide, followed by blocking of nonspecific binding with 1% bovine serum albumin (BSA) for 1 h at room temperature. The sections were then incubated overnight at 4 °C with an anti-PCNA mouse polyclonal antibody and an anti-CD31 mouse polyclonal antibody, respectively. After incubation with a goat anti-mouse IgG secondary antibody, the sections were incubated with peroxidase-labeled streptavidin for 1 h. Staining was visualized using a diaminobenzidine (DAB) solution, and the nuclei were counterstained with hematoxylin. Finally, representative images were captured under a microscope (PCNA-positive cells at 400× magnification; CD31-positive vessels at 200× magnification). The percentages of PCNA-positive cells and CD31-positive vessels within the images were analyzed.

### 4.7. Statistical Analysis

Data are presented as the mean ± standard deviation from three independent experiments (in vitro) and six replicates (in vivo). Statistical comparisons were performed using a two-tailed Student’s *t*-test assuming equal variance. A *p*-value of less than 0.05 was considered statistically significant.

## Figures and Tables

**Table 1:** The compositions of AP hydrogel precursor solutions with varying mass fractions of NaCit and CS.

Hydrogel Precursor Solution	AM	AA	DI	NaCit	NaAc	CS	BAM	Irgacure 2959
0% NaCit 0.5% CS	0.96 g	1 g	2.2 g	0 g	3 g	0.8 g	0.03 g	0.03 g
0.5% NaCit 0% CS	1 g	1 g	3 g	0.04 g	3 g	0 g	0.03 g	0.03 g
0.5% NaCit 0.25% CS	0.98 g	1 g	2.6 g	0.04 g	3 g	0.4 g	0.03 g	0.03 g
0.5% NaCit 0.5% CS	0.96 g	1 g	2.2 g	0.04 g	3 g	0.8 g	0.03 g	0.03 g
0.5% NaCit 0.75% CS	0.94 g	1 g	1.8 g	0.04 g	3 g	1.2 g	0.03 g	0.03 g
0.5% NaCit 1% CS	0.92 g	1 g	1.4 g	0.04 g	3 g	1.6 g	0.03 g	0.03 g
1% NaCit 0.5% CS	0.96 g	1 g	2.2 g	0.08 g	3 g	0.8 g	0.03 g	0.03 g

**Table 2:** Pressure generated by Von Frey filaments with different forces on hydrogel.

Von Frey Filament Force (g)	0.16	0.40	0.60	1.0	1.4	2.0
Pressure (kPa)	64.45	141.21	136.73	183.17	258.41	266.52
Von Frey Filament Force (g)	4	6	8	10	15	26
Pressure (kPa)	349.31	538.11	544.93	605.38	757.73	941.53

## Data Availability

The data presented in this study are available on request from the corresponding author.

## Supplementary Materials

The following supporting information can be downloaded at: https://www.mdpi.com/article/10.3390/gels12040347/s1, Figure S1: Chemical structural formulas of key components of PPTY-AP hydrogel; Figure S2: The effect of the PPTY layer on the thickness of the AP layer; Figure S3: Schematic illustration of component interactions in the PPTY-AP hydrogel; Figure S4: The gelation stability of AP hydrogel with different concentrations of CS; Figure S5: The compressive stress–strain relationship of PPTY-AP hydrogel; Figure S6: The antibacterial activity of hydrogel against *Escherichia coli* and *Staphylococcus aureus*.

## Abbreviations

The following abbreviations are used in this manuscript:

PPTY	The bottom layer
AP	The top armor protective layer
PAA	Polyacrylic acid
PAM	Polyacrylamide
TP	Tea polyphenol
YNBY	Yunnan Baiyao
PVA	Polyvinyl alcohol
NaAc	Sodium acetate
PPTY-AP	PPTY hydrogel and armor protective hydrogel
CS	Chitosan
NaCit	Sodium citrate
AP	Armor protective
BAM	N,N′-Methylenebis(acrylamide)
AM	Acrylamide
AA	Acrylic acid
