# Supplementary material for: A Multifunctional Shape-Adaptive Bilayer Hydrogel for Acute Hemostasis, Wound Repair, and Insect Bite Defense
Source: Gels. 2026 Apr 21;12(4):347. doi: 10.3390/gels12040347 (PMC13115842; doi:10.3390/gels12040347)
Supplement: Supplementary file 1 [file gels-12-00347-s001.zip › gels-4244832-supplementary.pdf]

Supplementary Material

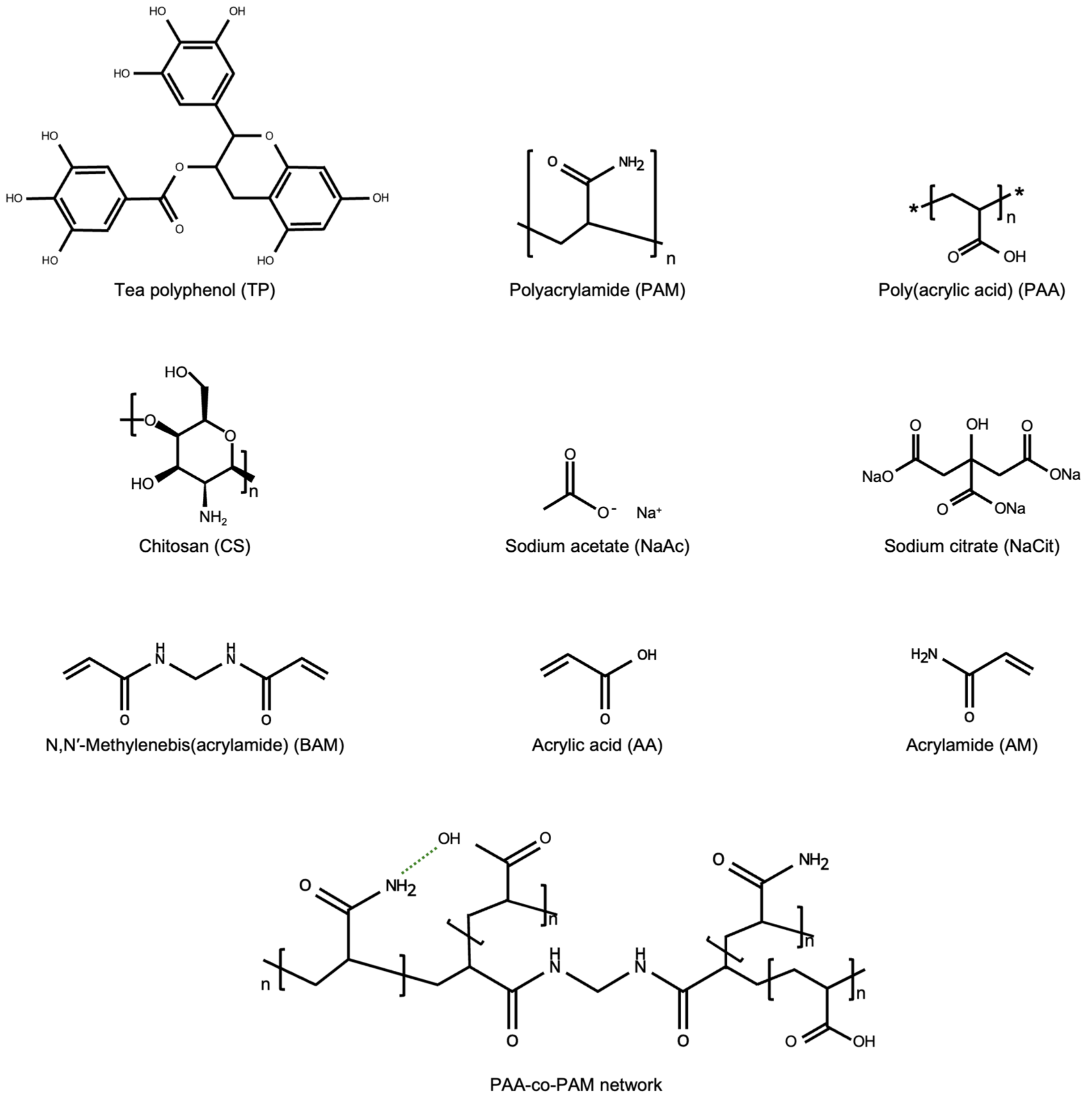

Figure S1. Chemical structural formulas of key components of PPTY-AP hydrogel.

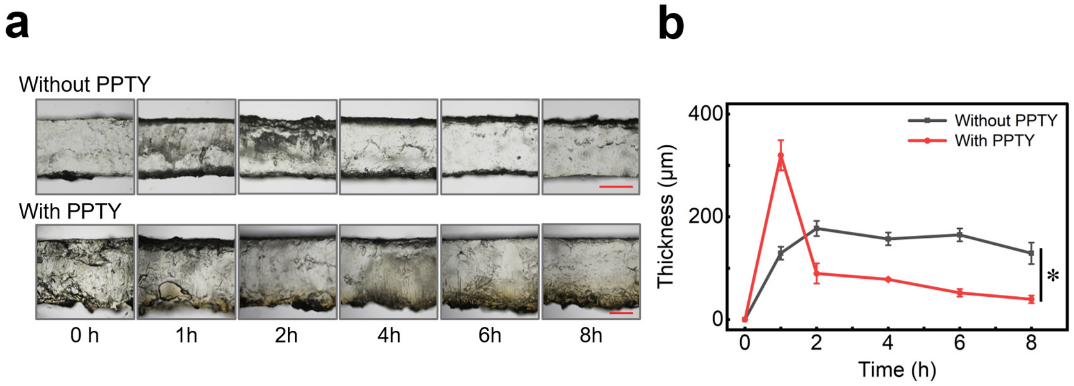

Figure S2. The effect of the PPTY layer on the thickness of the AP layer. (a) Images of AP layer hydrogel with and without PPTY layer, scale bar = 1 mm. (b) Quantitative representation of the thickness of armer layer.

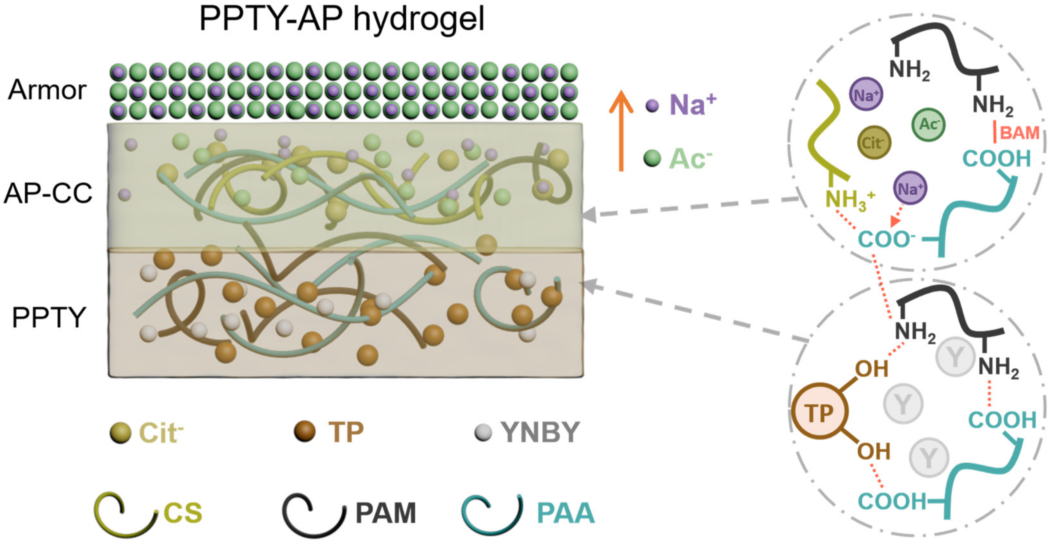

Figure S3. Schematic illustration of component interactions in the PPTY-AP hydrogel.

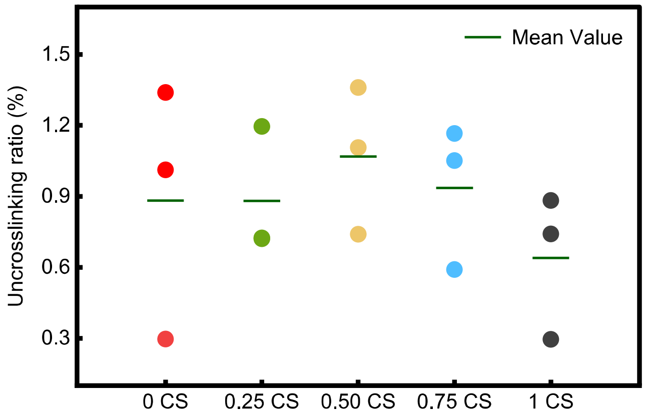

Figure S4. The gelation stability of AP hydrogel with different concentrations of CS.

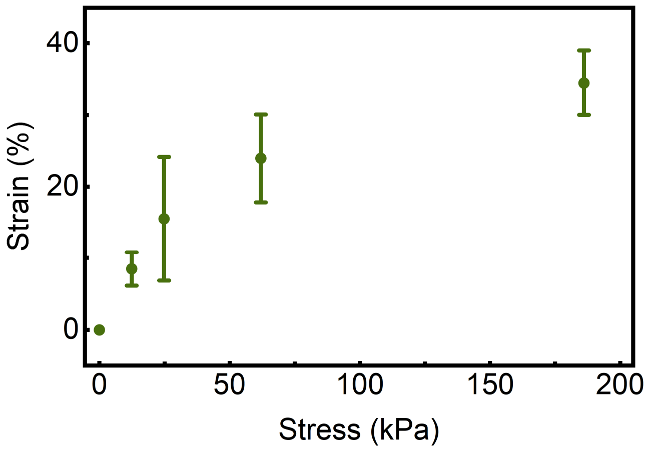

Figure S5. The compressive stress-strain relationship of PPTY-AP hydrogel.

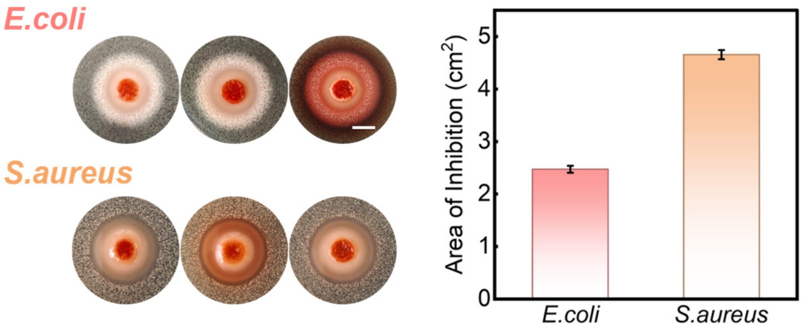

Figure S6. The antibacterial activity of hydrogel against *Escherichia coli* and *Staphylococcus aureus*. Scale bar = 1 cm.
